# Quorum sensing improves current output with *Acidithiobacillus ferrooxidans*

**DOI:** 10.1111/1751-7915.12797

**Published:** 2017-09-19

**Authors:** Nicolas Chabert, Violaine Bonnefoy, Wafa Achouak

**Affiliations:** ^1^ CEA, CNRS, UMR7265, ECCOREV FR 3098, LEMIRE, Laboratoire d'Ecologie Microbienne de la Rhizosphère et Environnement Extrêmes Aix Marseille Univ F‐13108 St Paul Les Durance France; ^2^ CNRS, Laboratoire de Chimie Bactérienne, Institut de Microbiologie de la Méditerranée Aix Marseille Univ Marseille France

## Abstract

*Acidithiobacillus ferrooxidans* is a strict acidophilic chemolithoautotrophic bacterium that obtains its energy from reduced inorganic sulfur species or ferrous iron oxidation under aerobic conditions. Carbon felt electrodes were pre‐colonized by *A. ferrooxidans* ATCC 23270^T^ using ferrous iron or sulfur as electron donors, via the addition (or not) of a mixture of C14 acyl‐homoserine lactones (C14‐AHLs). Electrode coverage during pre‐colonization was sparse regardless of the electron donor source, whereas activation of quorum sensing significantly enhanced it. Microbial fuel cells (MFCs) inoculated with pre‐colonized electrodes (which behaved as biocathodes) were more efficient in terms of current production when iron was used as an electron donor. Biocathode coverage and current output were remarkably increased to −0.56 A m^−2^ by concomitantly using iron‐based metabolism and C14‐AHLs. Cyclic voltammetry displayed different electrochemical reactions in relation to the nature of the electron donor, underlying the implication of different electron transfer mechanisms.

## Introduction

Microbial fuel cells (MFCs) are devices that convert chemical energy into electricity via the extracellular transfer of electrons by selected bacteria. While a wide range of natural environments (and therefore bacteria) can act as catalysts for anodic reactions (Chabert *et al*., 2015), only a limited number of species are known to catalyse cathodic oxygen reduction.

The extracellular electron transfer that allows bacteria to exchange electrons with an electrode is a natural phenomenon observed with conductive minerals (Shi *et al*., 2016). Indeed, bacteria such as *Geobacter* and *Schewanella* are known to catalyse anodic respiration. This ability relies on their capacity to reduce heavy metals when growing under anaerobic conditions. Therefore, it is expected that metal‐oxidizing bacteria could play a significant role in the construction of efficient biocathodes.

Ferrous iron oxidation occurs spontaneously in circumneutral aerobic environments (Roekens and Van Grieken, 1983), and iron‐oxidizing bacteria can be categorized into four physiological groups (Hedrich *et al*., 2011). Only one of these groups includes bacteria that are able to oxidize ferrous iron under high oxygen level conditions. Indeed, the spontaneous iron oxidation rate decreases under acidic conditions, indicating that acidophilic bacteria such as *Acidithiobacillus ferrooxidans* can oxidize iron under strict aerobic conditions. Moreover, as electrocatalytic reduction in O_2_ to H_2_O is kinetically limited by the availability of protons (Erable *et al*., 2009), *A. ferrooxidans* offers a great prospect for air biocathode design (Ter Heijne *et al*., 2007; Carbajosa *et al*., 2010; Rodrigues and Rosenbaum, 2014; Ishii *et al*., 2015).

*Acidithiobacillus ferrooxidans* is a strict acidophilic (pH 2) chemolithoautotrophic bacterium that obtains its energy from ferrous iron or reduced inorganic sulfur species oxidation under oxygenic conditions (Kelly and Wood, 2000). This bacterium is highly involved in biomining (Rawlings, 2002), and additional studies have thus been conducted on biofilm formation and quorum sensing in *A. ferrooxidans* and related species. Many biomining bacteria demonstrate acyl‐homoserine lactone (AHLs) communication (Ruiz *et al*., 2008; Bellenberg *et al*., 2014), and biofilm formation in the *A. ferrooxidans* strain ATCC 23270^T^ is more efficient on pyrite and sulfur coupons in the presence of added large acyl chains AHLs (Gonzalez *et al*., 2013; Mamani *et al*., 2016).

In the present study, we compared the impact of sulfur and ferrous iron oxidative pathways on the construction of efficient biocathodes with *A. ferrooxidans* ATCC 23270^T^. Furthermore, we have investigated the effect of C14‐AHLs on inert carbon electrode biofilm formation as well as current output. These results demonstrate that *A. ferrooxidans* ATCC 23270^T^ electroactivity could be effectively improved upon iron metabolism activation and by quorum sensing.

## Results and discussion

### C14‐AHLs improve biofilm formation

**Figure 1:**
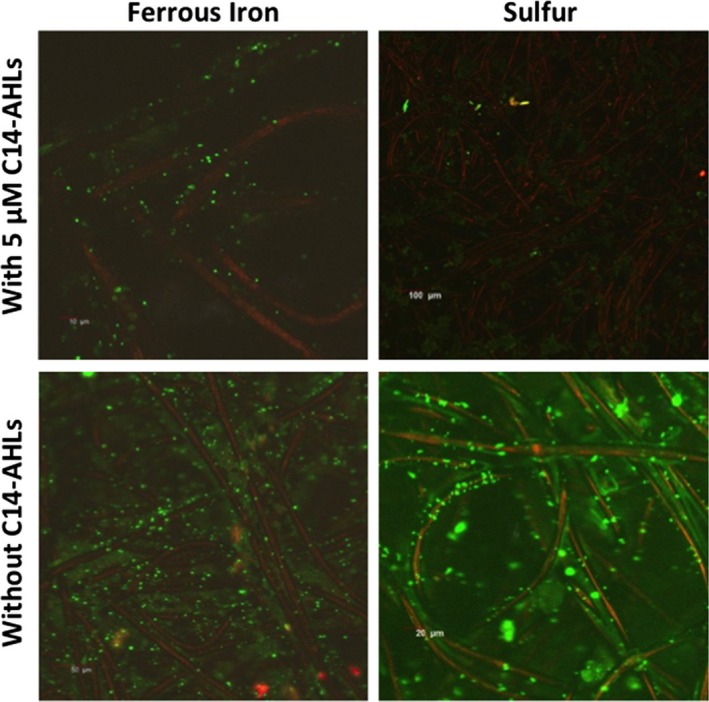
Confocal microscopy imaging of a carbon felt electrode after 7 days of culture. Cells labelled green using Syto^®^9 were imaged using an excitation at 489 nm and an emission at 510 nm. Carbon fibres were red‐imaged using an excitation at 645 nm and emission at 620 nm, in accordance with Rousseau *et al*., 2016. Electrode biofilms were formed by using ferrous iron (left column) or sulfur (right column) as energy sources, either with C14‐AHLs (lower panels) or without C14‐AHLs (upper panels).

*Acidithiobacillus ferrooxidans* is well known for its ability to colonize substrates such as pyrite, from which it obtains its energy by oxidizing iron or sulfur. Imaging colonized carbon felt electrodes revealed a low bacterial coverage (Fig. 1) when bacteria were cultivated for 1 week with ferrous iron (0.1 M) or sulfur (~2% *m*/*v*) as the energy source. Under these conditions, bacteria presumably favoured a planktonic lifestyle, because electron donors may not bind the unpolarized carbon felt electrode. As described by Gonzalez *et al*. (2013) for sulfur coupons and pyrite, *A. ferrooxidans* biofilm formation was highly improved by C14‐AHLs mixture containing C14‐AHL, 3‐hydroxy‐C14‐AHL and 3‐oxo‐C14‐AHL. When 5 μM of C14‐AHLs was added to cell culture on ferrous iron or on sulfur medium, *A. ferrooxidans* cells almost covered the whole electrode surface after 1 week (Fig. 1). Electrode colonization resulted in the adhesion of disparate cells to the carbon fibre in the form of a monolayer. No thick biofilms have ever been observed on pyrite with *A. ferrooxidans* (Vera *et al*., 2013).

### C14‐AHLs‐mediated biofilms improve current output

**Figure 2:**
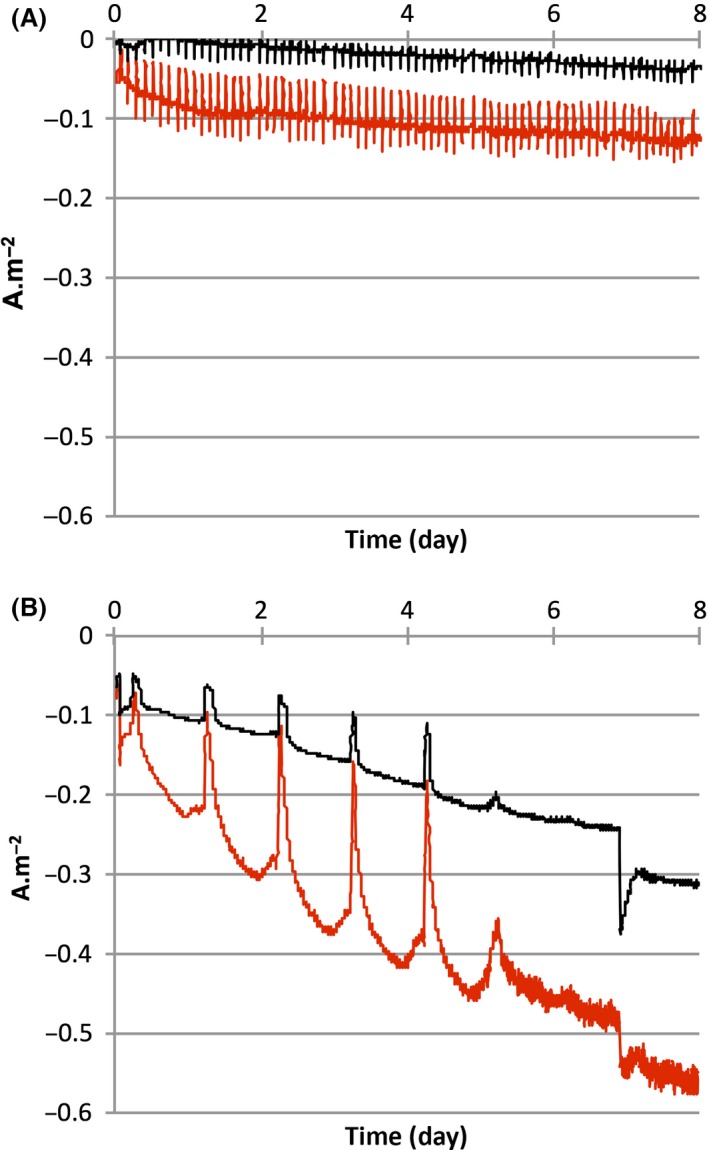
Chronoamperograms of 3 cm^2^ pre‐colonized carbon felt biocathodes. Sulfur (A) or ferrous iron (B) was used as the prior energy source, either with C14‐AHLs (red lines) or without C14‐AHLs (black lines). CA was performed on MPG‐2 potentiostat (Biologic) at *E* = −0.2 V versus Ag/AgCl KCl saturated reference and *T* = 30°C.

Several different mechanisms exist to produce electricity within MFCs. As current output involves a direct electron transfer resulting in a contact between the bacteria and the electrode, one of these mechanisms could potentially be biofilm‐dependent. To focus on this type of electron transfer with *A. ferrooxidans*, electricity production was monitored with ferrous iron or sulfur as the primary electron donor, with or without C14‐AHLs. When electrode pre‐colonization was performed with sulfur as the primary source of energy, current output only reached 0.05 A m^−2^ within 1 week (Fig. 2A). On the other hand, when electrode pre‐colonization occurred on ferrous iron, current output increased by approximately sixfold, with an intensity reaching −0.31 A m^−2^ (Fig. 2B) within 1 week. Our results show that C14‐AHLs increase electrode colonization, indicating the possibility of a better current output. Chronoamperometry (CA) data confirmed the current output increase using a higher electrode coverage: when 5 μM of C14‐AHLs was added during electrode colonization, the current intensity reached −0.12 (Fig. 2A) and −0.56 A m^−2^ (Fig. 2B) for sulfur and ferrous iron as the primary energy sources respectively.

Subsequently, cyclic voltammetry (CV) was performed after CA to determine how colonization, sulfur‐based or iron‐based cell metabolism, and C14‐AHLs affect electrochemical reactions.

### Sulfur‐based metabolism

**Figure 3:**
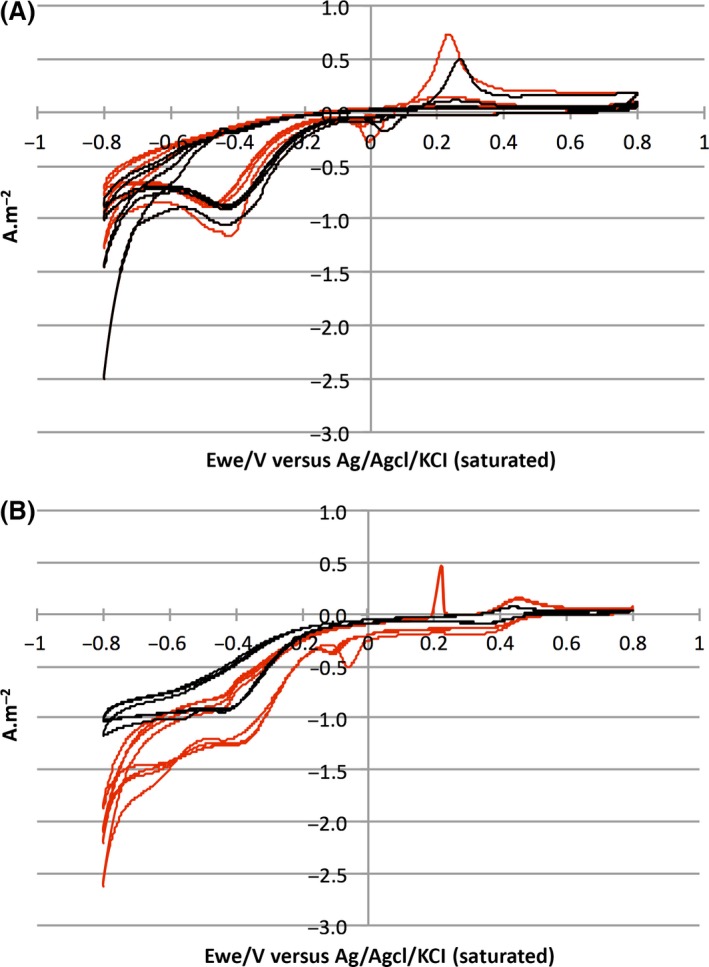
Cyclic voltammetry plots for five consecutive cycles of biocathodes. Electrodes were formed by using either sulfur (A) or ferrous iron (B) as the prior energy source, either with C14‐AHLs (red lines) or without C14‐AHLs (black lines). CV curves were obtained at a scan rate of 5 mV s^−1^.

Electrodes colonized with sulfur as the primary energy source showed slight differences, whether they had been formed with C14‐AHLs or not (Fig. 3A). Indeed, CV with and without C14‐AHLs displayed redox reactions that occur at a positive potential but disappear after the first cycle. However, the peak intensities were somewhat higher when the electrodes were formed with C14‐AHLs. This same pattern was observed for the reaction at −0.44 V versus the reference. Specifically, after the first cycle, the intensity decreased but remained slightly higher than −1.14 A m^−2^ when the electrode was formed in the presence of C14‐AHLs, whereas the maximum was −1.04 A m^−2^ in the absence of C14‐AHLs. Nevertheless, when C14‐AHLs were used during electrode colonization, redox reactions occurring at the positive potential had a tendency to be driven at a lower potential. This is presumably linked to the higher coverage of the electrode surface.

### Iron‐based metabolism

When ferrous iron was used as the primary energy source, significant differences were noticed relative to the quorum sensing response and the nature of the primary energy source (Fig. 3B). CV performed on electrodes formed without C14‐AHLs only showed a reduction at −0.4 V versus the reference, reaching −0.82 A m^−2^. In contrast, at least three reactions occurred upon quorum sensing activation during colonization of the electrode. Two of these reactions were likely the same as those observed on sulfur with an oxidation at 0.2 V versus the reference, whereas the third reaction corresponded to a reduction occurring around 0 V versus the reference. Therefore, the oxidation peak was stable over the five cycles. However, activation of quorum sensing during colonization with ferrous iron as the primary energy source affected the reduction occurring around −0.4 V versus the reference. Specifically, the reduction intensity ranged from −0.82 to −1.22 A m^−2^ and the potential increased from −0.4 V to −0.34 V versus the reference for pre‐colonized electrodes without and with C14‐AHLs respectively. This emphasizes the effect of electrode colonization and the potential of *A. ferrooxidans* ATCC 23270^T^ to drive oxygen reduction using the cathode as electron donors. These results demonstrate that a well‐colonized electrode tends to decrease the redox potential of the reaction. Furthermore, when quorum sensing was activated, the current output was nearly increased by about twofold, whether the electrode was formed with sulfur or ferrous iron as the primary energy source.

### Mechanisms underlying electron transfer

The twofold increase in current output confirms that extracellular electron transfer of *A. ferrooxidans* is linked to cell adhesion on the cathode, because current increase was observed when electrodes were formed with quorum sensing activation. Nonetheless, differences in current intensity cannot be due exclusively to electrode coverage. When quorum sensing was activated, electrodes formed with either sulfur or ferrous iron displayed a similar colonization. Quorum sensing acts at the gene level and may activate other genes than those involved in biofilm formation, resulting in improved current output. Moreover, *A. ferrooxidans* does not utilize the same metabolic pathway to metabolize sulfur or ferrous iron (Quatrini *et al*., 2009). The main difference occurs at the beginning of the respiratory chains: electron uptake occurs at the cytoplasmic membrane for sulfur oxidation and at the outer membrane for ferrous iron oxidation. Indeed, when ferrous iron acts as an electron donor, a *c*‐type cytochrome (Cyc2) is involved in electron uptake (Yarzábal *et al*., 2002). Furthermore, a higher number of *c*‐type cytochromes are found in *A. ferrooxidans* when it is cultivated on ferrous iron as opposed to sulfur (Yarzábal *et al*., 2002). Finally, it was previously shown that *c*‐type cytochromes are involved in extracellular electron transfer (Rosenbaum *et al*., 2011; Kumar *et al*., 2016), suggesting that Cyc2 may be responsible for improving the efficiency of electricity production in *A. ferrooxidans* ATCC 23270^T^ when the electrodes are pre‐colonized on ferrous iron.

## Conclusions

Quorum sensing activation using C14‐AHLs remarkably increased electrode colonization by *A. ferrooxidans* ATCC 23270^T^, resulting in a twofold enhancement of current output. We have shown that prior activation of iron metabolism leads to a higher electroactivity in this strain. This phenomenon could be directly linked to the ability of metal‐oxidizing bacteria to take up electrons from a solid surface, whereas other examples such as sulfur metabolism occurs internally. Further investigations, such as the transcriptomic analysis of electrode‐colonizing bacteria, are required to elucidate the extracellular electron transfer pathway involved in the electroactivity of strain ATCC 23270^T^.

## Conflict of interest

The authors declare that they have no conflict of interests.
